# Aqueous Extracts of Fermented Macrofungi Cultivated in Oilseed Cakes as a Carbon Source for Probiotic Bacteria and Potential Antibacterial Activity

**DOI:** 10.3390/metabo13070854

**Published:** 2023-07-18

**Authors:** Joice Raísa Barbosa Cunha, Daiana Wischral, Rubén Darío Romero Peláez, Pérola De Oliveira Magalhães, Marina Borges Guimarães, Maria Aparecida de Jesus, Ceci Sales-Campos, Thais Demarchi Mendes, Eustáquio Souza Dias, Simone Mendonça, Félix Gonçalves de Siqueira

**Affiliations:** 1 Embrapa Agroenergia, Distrito Federal, Brasília 70770-901, Brazilmarinaborgesguimaraes@gmail.com (M.B.G.);; 2 Programa de Pós-Graduação em Microbiologia Agrícola, Universidade Federal de Lavras (UFLA), Lavras 37200-000, Brazil; 3 Instituto de Ciências Biológicas, Universidade Federal de Brasília, Brasília 70910-900, Brazil; 4 Departamento de Farmácia, Faculdade de Ciências da Saúde, Universidade Federal de Brasília, Brasília 70910-900, Brazil; 5 Instituto Nacional de Pesquisas da Amazônia (INPA), Manaus 69067-375, Brazil; ranna@inpa.gov.br (M.A.d.J.);

**Keywords:** antimicrobial activities, biodetoxification, fermentation, macrofungi bioactive, oil seed cake

## Abstract

Plant biomass colonized by macrofungi can contain molecules with bioactive properties with applications to human/animal health. This work aimed to verify antibacterial activities from aqueous extracts from oil seed cakes of *Jatropha curcas* (JSC) and cottonseed (CSC), fermented by macrofungi for probiotic bacteria cultivation. *Coriolopsis* sp., *Tyromyces* sp., *Panus lecomtei*, and *Pleurotus pulmonarius* were cultivated in solid and submerged media. The aqueous extract of unfermented JSC was more efficient than glucose for the growth of all probiotic bacteria. Extracts from four macrofungi fermented in CSC favored *Lactobacillus acidophilus* growth. In solid fermentation, macrofungi extracts cultivated in JSC favored *Bifidobacterium lactis* growth. All fungi extracts showed more significant growth than carbohydrates among the four probiotic bacteria evaluated. Regarding antimicrobial activities, no fungal extract or bacterial supernatant showed a more significant inhibition halo for enteropathogenic bacteria than ampicillin (control). Extracts from *P. lecomtei* and *Coriolopsis* sp. in CSC showed inhibition halos for *Salmonella enterica*. Supernatants from *L. acidophilus*, *B. lactis*, and *Lactobacillus rhamnosus* resulted in more significant inhibition of *Staphylococcus aureus* than the control, which indicates possible antimicrobial activity. Unfermented JSC supernatant showed better results for bacterial growth, while supernatants and aqueous extracts from CSC fermentation can be used for probiotic bacteria culture.

## 1. Introduction

Mushrooms are known to be rich in antioxidant compounds with anti-inflammatory and anticancer activities [1] and complex carbohydrates that behave like intestinal fibers and can help in the growth of probiotic microorganisms and the production of short-chain fatty acids that favor these microorganisms over enteropathogens [2].

The most commonly bioactive compounds found in mushrooms include polysaccharides [3,4], proteins [5], terpenes [6,7], unsaturated fatty acids [8], and phenolic compounds [9,10]. In mushrooms, these compounds provide antioxidant, antimicrobial, antitumor, anti-inflammatory, antidiabetic, and hypocholesterolemic activities, among other important biological activities [10,11].

More than 400 species of bacteria are in the intestine, forming a complex and essential microbiota mainly composed of the genera *Bifidobacteria* and *Lactobacillus* [12], Gram-positive bacteria that ferment carbohydrates into acetic and lactic acids, respectively [13]. Some intestinal bacteria are homofermentative because they ferment glucose only into lactic acid, and others are heterofermentative because they ferment hexoses and pentoses via phosphoketolase, obtaining acetic acid, CO_2_, and ethanol [14]. Probiotics can improve feed conversion to nutrients, regulate the immune response, inhibit pathogen growth, stimulate beneficial microbiota, and form biofilms to block infections [15].

These *Bifidobacteria* and *Lactobacillus* are known as probiotics, as they suppress the growth of enteropathogenic bacteria, improve food absorption and gastrointestinal tract function, and are also capable of modulating the immune system [16]. Prebiotics are non-digestible food components that selectively stimulate the growth of these probiotic bacteria that, in addition to modulating the intestinal microbiota, attenuate health conditions such as diabetes, obesity, and cancer [17].

In addition to probiotic bacteria, other bacteria may be present in the gastrointestinal tract, and some may be pathogenic, causing harm to hosts. Bacteria such as *Salmonella* sp., *Campylobacter*, *Escherichia coli*, *Listeria* monocytogenes, *Clostridium botulinum*, Microsporidia, and *Campylobacter jejuni* are frequently associated with intestinal infections [18,19]. However, the inappropriate use of antibiotics over the years has led to resistant bacteria, which has been an increasing threat to health. Researchers from all around the world are now focused on finding alternative antimicrobials, and the chemical composition of some mushrooms suggests that they may have antiviral and antibacterial potential [20,21].

The present work aimed to evaluate the prebiotic (carbon sources of probiotic bacteria) and antibacterial potential of supernatants from submerged fermentation (SmF) and aqueous extracts from solid-state fermentation (SSF) of residues from cakes of seed oil cotton and *Jatropha* by *Coriolopsis* sp. INPA1646, *Tyromyces* sp. INPA1696, *P. lecomtei* BRM 044603, and *P. pulmonarius* BRM 055674.

## 2. Materials and Methods

### 2.1. Obtaining Supernatants and Extracts

*Coriolopsis* sp. INPA1646, *Tyromyces* sp. INPA1696, *P. lecomtei* BRM 044603, and *P. pulmonarius* BRM 055674 belong to the INPA Macrofungi Collection (Manaus/AM) and the Microorganisms and Microalgae Collection applied to the biorefinery of Embrapa Agroenergia—CMMABio (Braslia/DF). The strains were reactivated on plates with PDA (Potato Dextrose Agar) and incubated at 28 °C for 15 days.

Submerged fermentation (SmF) was performed in flasks with 5.0 g of each dry biomass and 100 mL of distilled water, and solid-state fermentation in flasks with 40 g of JSC or 20 g of dry CSC, with moisture adjusted to approximately 70%. After sterilization, the liquid medium flasks were inoculated with 10 mycelial plugs (8 mm in diameter) of each fungus and incubated at 28 °C and 180 rpm for 14 days. The solid culture flasks with 10 plugs in the JSC flasks and 5 in the CSC flasks were incubated at 28 °C for 14 days. Controls consisted of sterilized biomass under the same conditions without fungal inoculation.

After incubation, submerged cultures were vacuum filtered to obtain supernatants, and the solid-state fermented cultures were dried at 60 °C, crushed, and subjected to aqueous extraction under pressure and temperature in an ASE—Accelerated Solvent Extractor (Dionex ASE 350). Supernatants and aqueous extracts were frozen and lyophilized.

### 2.2. Determination of Prebiotic Activity

The potential prebiotic activity (carbon sources of probiotic bacteria) was evaluated by the ability of supernatants and aqueous extracts to stimulate the growth of *Lactobacillus acidophilus*, *Lactobacillus plantarum*, *Lactobacillus rhamnosus*, and *Bifidobacterium lactis* strains in MRS (−C) medium (10.0 g/L of peptone + 10.0 g/L of meat extract + 5.0 g/L of yeast extract + 1.0 g/L of polysorbate 80 + 2.0 g/L of ammonium citrate + 5.0 g/L of sodium acetate + 0.1 g/L of magnesium sulfate + 0.05 g/L of manganese sulfate + 2.0 g/L of monopotassium phosphate) for 48 h at 37 °C. The strains, provided by the Central Public Health Laboratory of the Federal District (Lacen-UnB), were reactivated in MRS Broth, and diluted in 0.9% saline solution to obtain a 10^6^ CFU/mL concentration according to the McFarland standard.

The supernatants and aqueous extracts were diluted in sterile distilled water at a concentration of 40 mg/mL, filtered through a 0.22 µm membrane, and added to MRS Broth without glucose and twice the concentration of the other components (2×MRS-C) and 0.4% L-cysteine in a 1:1 (*v*:*v*) ratio. Three positive controls were performed: glucose (Sigma-Aldrich, Merck Millipore, Germany), fructooligosaccharides (FOS) at 90% purity (Newnutrition, Ribeirão Preto, SP, Brazil), and inulin from chicory (FiberBem, The Netherlands) at 20 mg/mL. The negative control consisted of sterile distilled water.

Bacterial growth was monitored in 2 mL of medium with 5 µL of inoculum and then transferred to two 96-well microplates (Elisa plates), 200 µL per well, in triplicate, and sterile controls consisting of wells with MRS medium without inoculum. The plates were sealed with highly translucent 2.0 polyethylene Platemax sealing membranes for PCR transport and storage (Axygen^®^ Corning Inc., Corning, NY, USA) and incubated at 37 °C and 180 rpm for 48 h, with absorbption readings at 600 nm (OD 600) every hour for the first 12 h and then every 2 h for 48 h in total.

Viable cells were quantified in 100 µL of each treatment and collected at 12, 24, and 48 h of incubation at 37 °C for serial dilution and plating by spreading on plates of complete MRS medium with 0.2% L-cysteine, and colonies were counted after 48 h of incubation. The growth of total cells was represented by the change in OD and the growth of viable cells in Petri dishes.

Elisa plates, with the remaining 800 µL of the medium, were also incubated in a shaker at 180 rpm and 37 °C for 48 h to obtain the supernatants by centrifugation at 10,000 rpm for 15 min, which were also tested in the antimicrobial activity assays.

### 2.3. Determination of Antimicrobial Activity

*Escherichia coli* (ATCC 25922), *Salmonella enterica* subsp. *Enterica serovar Typhimurium* (ATCC 14028), and *Staphylococcus aureus* (ATCC 27154, enterotoxin-producing strain) from the Natural Products Laboratory of the University of Brasilia (UnB) were reactivated in Mueller–Hinton Agar at 37 °C for 48 h. Bacteria were grown for 24 h at 37 °C and 180 rpm in Mueller–Hinton broth and inoculated by spreading on Mueller–Hinton Agar plates for an antibiogram. Supernatants and dry aqueous extracts were diluted in sterile distilled water at a concentration of 100 mg/mL and filtered through a 0.22 µm membrane. Autoclaved paper discs were prepared with 20 µL of the samples, their dilutions (1:10, 1:100, and 1:1000), and the supernatants from the above-mentioned prebiotic bacteria growth. After room temperature drying, the discs were placed on the plates with the microorganisms and incubated at 35 °C for 24 h to assess the formation of inhibition halos. Ampicillin discs were used as a positive control (10 μg) [22].

### 2.4. Statistical Analysis 

Experiments were performed in triplicate, and the results were submitted to the Shapiro–Wilk normality test and Tukey’s test with a significance level of 5% (*p* < 0.05) with SISVAR 5.6 software [23].

## 3. Results

### 3.1. Prebiotic Activity

#### 3.1.1. *Lactobacillus acidophilus*

**Figure 1:**
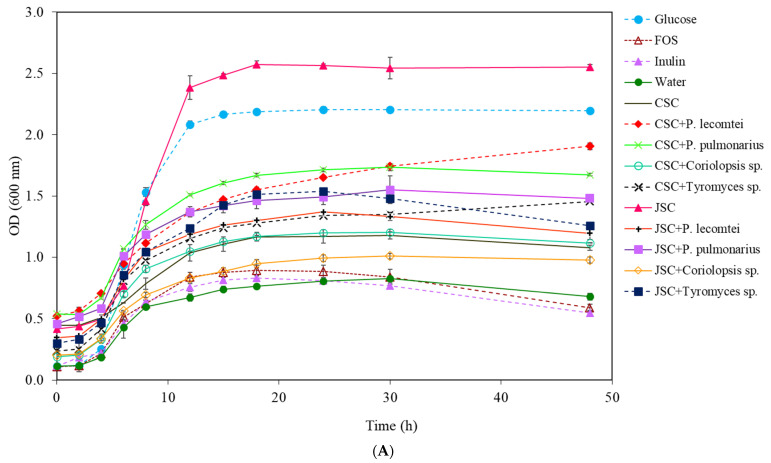

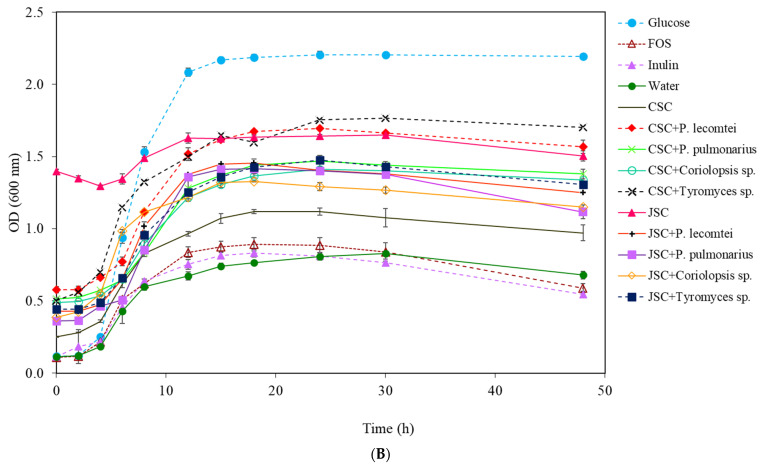
Growth curves of *L. acidophilus* at 20 mg/mL for 48 h at 37 °C: (**A**) supernatants (carbon source—prebiotics) from submerged fermentations by macrofungi in cottonseed cake (SmF-CSC) and jatropha seed cake (SmF-JSC); (**B**) supernatants from solid-state fermentation by macrofungi in cottonseed cake (SSF-CSC) and jatropha seed cake (SSF-JSC).

The highest increase of total cells of *L. acidophilus* was observed in MRS medium (−C) with 20 mg/mL of supernatant SmF-JSC incubated for 14 days at 28 °C without fungus (Figure 1A), a variation of 2.139 ± 0.022 A (Table 1), followed by medium containing glucose (2.081 ± 0.017 A). The positive controls, FOS, and inulin, provided similar results to the negative control (water), just as Rodrigues et al. (2016) observed in *Lactobacillus* sp. [24]. In the treatments of JSC fermented by fungi, the highest growth of *L. acidophilus* was obtained in the medium of *P. pulmonarius* BRM 055674 supernatant with 1.022 ± 0.015 A after 48 h of incubation, followed by the supernatants of *Tyromyces* sp. INPA1696 (0.960 ± 0.058 A), *P. lecomtei* BRM 044603 (0.849 ± 0.053 A), and *Coriolopsis* sp. INPA1646 (0.774 ± 0.028 A). 

After 14 days of cultivation, supernatants of SSF fermentation by *P. lecomtei* BRM 044603, *Tyromyces* sp. INPA1696, and *P. pulmonarius* BRM 055674 resulted in more significant growth of *L. acidophilus* than unfermented SmF supernatant, probably due to the release of fermentable sugars (Figure 1B). Likewise, the SSF supernatant fermented by *Coriolopsis* sp. INPA1646 showed more significant total cell growth (0.922 ± 0.009 A) than the unfermented CSC supernatant (0.630 ± 0.026 A) and controls; however, its growth was lower when compared to the glucose treatment.

The variation in OD of total *L. acidophilus* cell growth at the end of 48 h of incubation allows us to conclude that the best culture medium was MRS (−C) with 20 mg/mL of JSC supernatant incubated for 14 days at 28 °C without fungal fermentation, having better results than the conventional MRS medium (with 20 mg/mL of glucose). 

The growth results of *L. acidophilus* in the aqueous extracts from the solid-state fermentation of CSC were overall lower than those observed using the supernatants from the submerged fermentation, as seen in Table 1, which shows the variations in optical densities before and after 48 h of incubation. In this group, the highest growth of *L. acidophilus* was observed in the aqueous extract of SSF fermented by *Tyromyces* sp. INPA1696, followed by the CSC fermented by *P. lecomtei* BRM 044603. The growth of *L. acidophilus* in the aqueous extract of JSC without fungus was the lowest observed among all samples tested. The opposite was observed with the JSC supernatant; this suggests that the treatment and extraction, with agitation and 28 °C after 14 days, are more efficient than the aqueous extraction, under pressure and temperature in ASE, to obtain *L. acidophilus* culture medium, probably due to the release of fermentable sugars.

**Figure 2:**
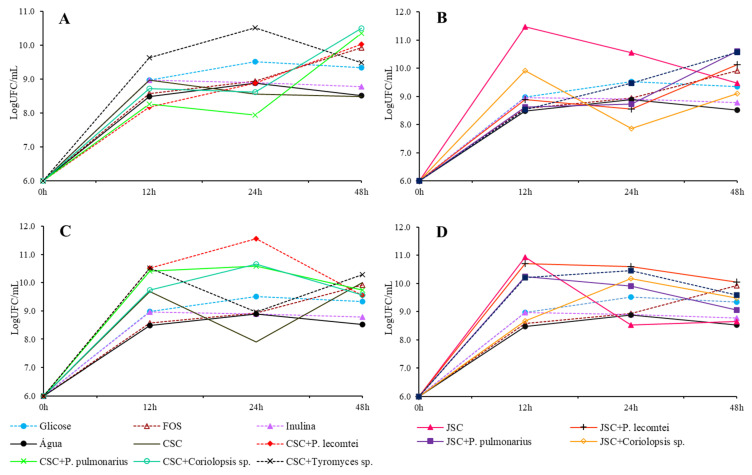
Growth of *L. acidophilus* in LogUFC/mL with 0, 12, 24, and 48 h of incubation in MRS-C medium with 20 mg/mL of CSC submerged fermentation supernatants: (**A**), submerged fermentation supernatants of JSC (**B**), solid-state fermentation aqueous extracts of CSC (**C**), and solid-state fermentation extracts of JSC (**D**).

The peak of total cells in the medium does not always occur in the 48th hour of incubation. However, maintaining cell viability for long periods of time is crucial to consider as a prebiotic activity. The highest concentration of viable *L. acidophilus* cells is observed at the 12th hour of incubation in the medium containing the unfermented JSC supernatant (Figure 2B). Among the submerged CSC fermentation supernatants (Figure 2A), the highest concentration of viable cells was observed in the fermented medium with *Tyromyces* spp. INPA1696 after 24 h of incubation, *P. pulmonarius* BRM 055674, and *Coriolopsis* sp. INPA1646 after 48 h of incubation. For the solid CSC fermentation aqueous extracts (Figure 2C), the highest concentration occurs with the extract of *P. lecomtei* BRM 044603 at 24 h of incubation. In JSC treatments (Figure 2B,D), none surpasses the concentration in the medium with unfermented JSC supernatant after 12 h of incubation. However, the extract of solid fermentation by *P. lecomtei* BRM 044603 equals this concentration in 12 h of cultivation.

#### 3.1.2. *Bifidobacterium lactis*

**Figure 3:**
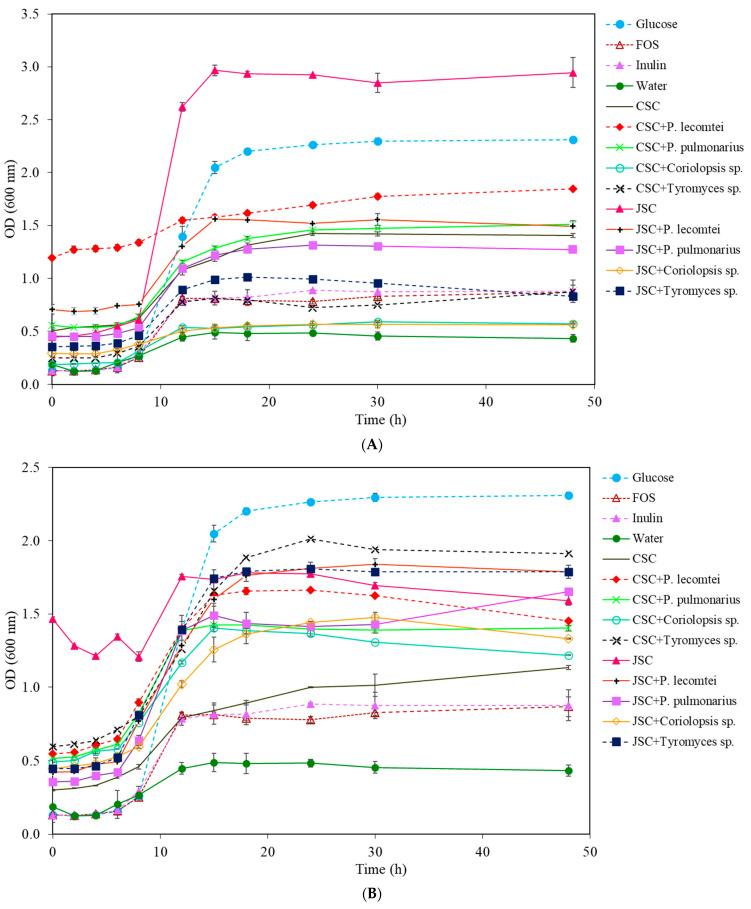
Growth curves of *B. lactis* at 20 mg/mL for 48 h at 37 °C: (**A**) supernatants from submerged fermentations by macrofungi in cottonseed cake (SmF-CSC) and jatropha seed cake (SmF-JSC); (**B**) supernatants from solid-state fermentation by macrofungi in cottonseed cake (SSF-CSC) and jatropha seed cake (SSF-JSC).

In *B. lactis* cultures, the commercial prebiotics FOS and inulin did not show satisfactory growth. The peak of growth in the medium with glucose was observed in the 15th hour of incubation (Figure 3A). The most significant growth of whole-cell *B. lactis* was also observed in the supernatant medium with JSC without fungal inoculation, with an increase of 2.501 ± 0.140 A after 48 h of incubation, followed by the glucose treatment with 2.122 ± 0.086 A.

In cultures with supernatants obtained from CSC, the most significant cell growth after 48 h of incubation was observed in the treatment fermented by *P. pulmonarius* BRM 055674, with an increase in OD of 0.949 ± 0.059 A, followed by the CSC supernatant without fungus, with an OD of 0.907 ± 0.012 A (Table 2). The supernatant from CSC fermentation by *P. lecomtei* (BRM 044603) showed an OD variation of 0.654 ± 0.003 A. However, the dark color of the assay may interfere with the absorbance reading and make it difficult to quantify the increase in total cells. The supernatant from the fermentation by *Tyromyces* sp. INPA1696 behaved similarly to the positive controls FOS and inulin in the *B. lactis* growth; the treatment with *Coriolopsis* sp. INPA1646 was similar to the negative control (water), with an OD of 0.379 ± 0.007 A (Figure 3B).

On the other hand, in cultures with aqueous extracts obtained from CSC, the treatment with *Tyromyces* sp. INPA1696 showed an OD variation of 1.318 ± 0.087 A after 48 h of incubation (Table 2). The treatment with CSC extract without fungus was similar to that observed in the media with CSC extracts fermented by *P. pulmonarius* BRM 055674 and *P. lecomtei* BRM 044603, while the absorbance in the medium with CSC extract fermented by *Coriolopsis* sp. INPA1646 was only 0.728 ± 0.005 A.

In cultures with supernatants obtained with JSC, treatments fermented by *P. lecomtei* BRM 044603 and *P. pulmonarius* BRM 055674 showed similar results in stimulating the growth of *B. lactis* and were higher when compared to the positive controls FOS and inulin. In cultivations with aqueous extracts, the treatments of JSC fermented by *P. lecomtei* BRM 044603, *P. pulmonarius* BRM 055674, and *Tyromyces* sp. INPA1696 showed greater growth stimulation of *B. lactis* (Table 2). 

**Figure 4:**
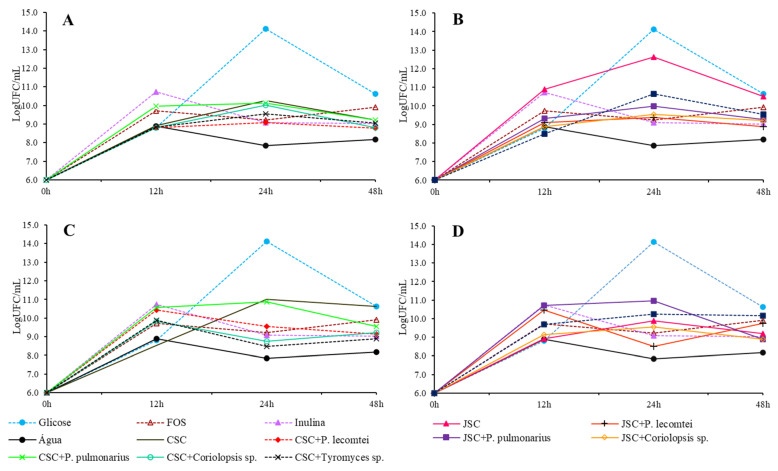
Growth of *B. lactis* in LogUFC/mL with 0, 12, 24, and 48 h of incubation in MRS-C medium with 20 mg/mL of CSC submerged fermentation supernatants (**A**), submerged fermentation supernatants of JSC (**B**), solid-state fermentation aqueous extracts of CSC (**C**), and solid-state fermentation extracts of JSC (**D**).

The highest concentrations of viable cells occurred after 24 h of incubation in the medium with glucose (conventional MRS), followed by the medium containing the unfermented JSC supernatant. However, if shorter incubation times were considered (e.g., 12 h), the media with the supernatants and biomass extracts had higher concentrations of viable cells (Figure 4).

#### 3.1.3. *Lactobacillus rhamnosus*

**Figure 5:**
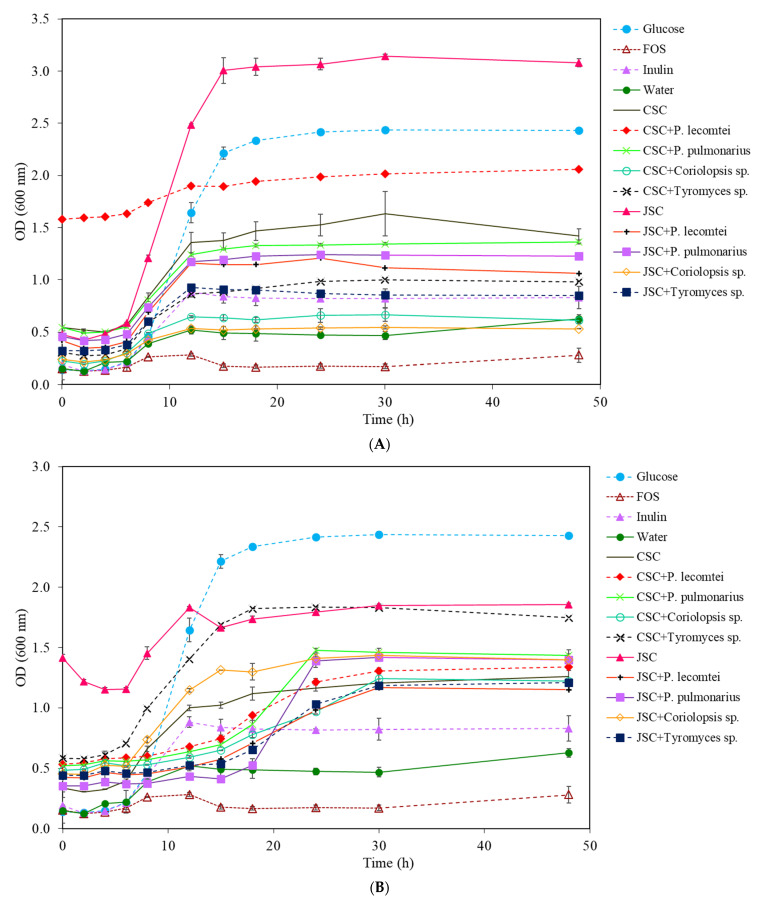
Growth curves of *L. rhamnosus* at 20 mg/mL for 48 h at 37 °C: (**A**) supernatants from submerged fermentations by macrofungi in cottonseed cake (SmF-CSC) and jatropha seed cake (SmF-JSC); (**B**) solid-state fermentation by macrofungi in cottonseed cake (SSF-CSC) and jatropha seed cake (SSF-JSC).

The growth of *L. rhamnosus* was strongly stimulated using JSC supernatant without fungus, reaching the exponential phase in a shorter time (6–12 h) (Figure 5A), with an increase in OD of 2.570 ± 0.057 A at the end of 48 h of incubation, higher than the control with glucose (OD 2.279 ± 0.025 A). Among the JSC supernatants tested, treatment with *Coriolopsis* sp. INPA1646 showed lower growth, with an increase in OD of 0.302 ± 0.032 A at the end of 48 h of incubation.

The results of this bacterium growth in the aqueous extracts obtained from solid-state fermentation with macrofungi did not have the same pattern as those observed in the supernatants (Figure 5B), with the lowest growth in the JSC extract without fungus (0.397 ± 0.091 A) and more significant growth in the extract of CSC fermented by *Tyromices* sp. INPA1696 (1.199 ± 0.091 A), followed by JSC extract fermented by *P. pulmonarius* BRM 055674 (1.042 ± 0.049 A).

**Figure 6:**
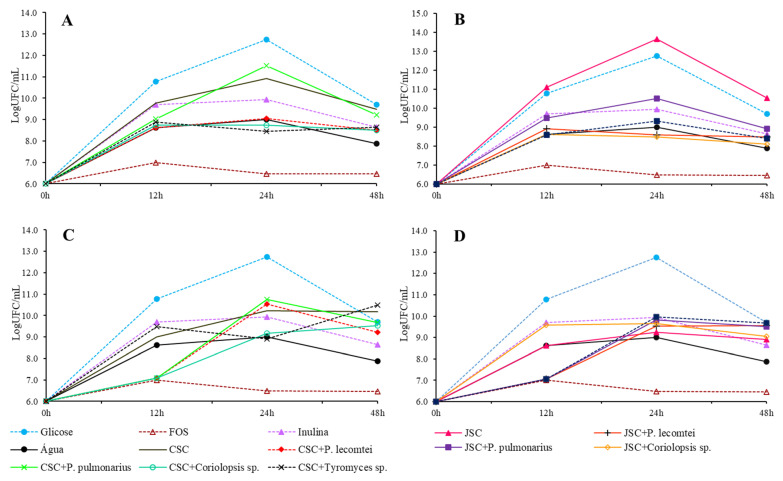
Growth of *L. rhamnosus* in LogUFC/mL with 0, 12, 24, and 48 h of incubation in MRS-C medium with 20 mg/mL of CSC submerged fermentation supernatants (**A**), submerged fermentation supernatants of JSC (**B**), solid-state fermentation aqueous extracts of CSC (**C**), and solid-state fermentation extracts of JSC (**D**).

In the cultures with CSC supernatants, growth of *L. rhamnosus* showed more significant variation in the treatment without fungus, with an OD of 0.881 ± 0.059 A. Among the fermented treatments, the highest growth value was observed in the treatment with *P. pulmonarius* BRM 055674 (0.825 ± 0.002 A), followed by *P. lecomtei* BRM 044603 (0.481 ± 0.003 A) and *Coriolopsis* sp. INPA1646 (0.404 ± 0.081 A) (Table 3). In aqueous extracts of CSC, the treatments fermented by *Tyromyces* sp. INPA1696 and *P. pulmonarius* BRM 055674 showed more significant growth of *L. rhamnosus* after 48 h than the aqueous extract of CSC without fungus. The highest concentration and viable cells were found after 24 h of incubation in the medium with 20 mg/mL of unfermented JSC supernatant and in the medium with glucose (conventional MRS) with 24 h of incubation as well (Figure 6).

#### 3.1.4. *Lactobacillus plantarum*

**Figure 7:**
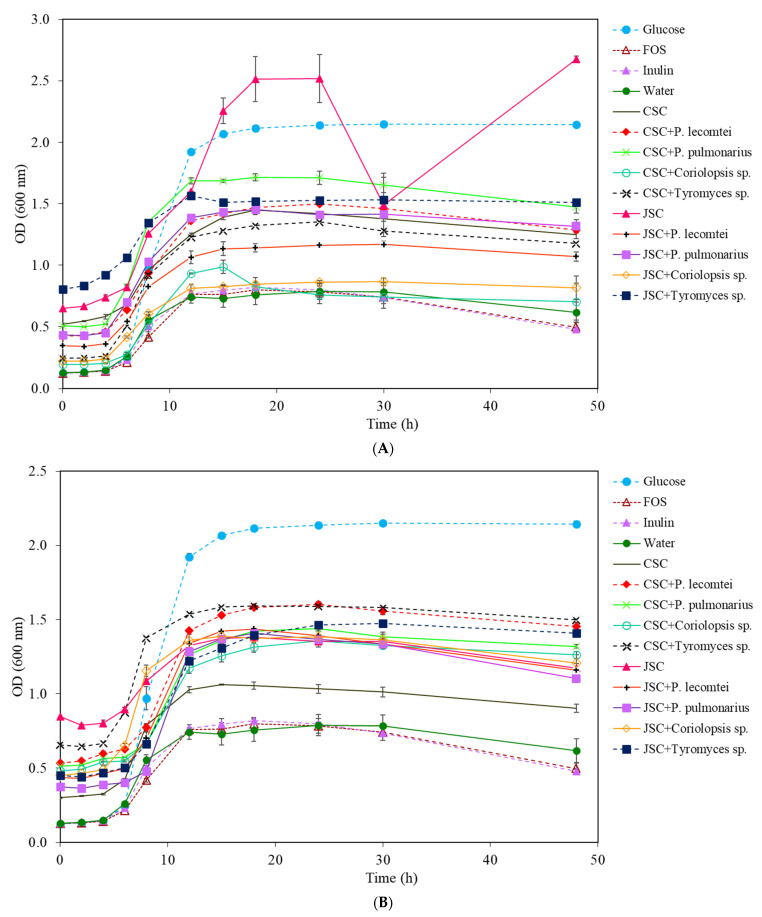
Growth curves of *L. plantarum* at 20 mg/mL for 48 h at 37 °C: (**A**) supernatants from submerged fermentations by macrofungi in cottonseed cake (SmF-CSC) and jatropha seed cake (SmF-JSC); (**B**) supernatants from solid-state fermentation by macrofungi in cottonseed cake (SSF-CSC) and jatropha seed cake (SSF-JSC).

The growth of the bacteria *L. plantarum* had a significant stimulus, indicated by the variation of OD, using MRS media with glucose (2.014 ± 0.018 A) and the supernatant JSC without fungus (2.026 ± 0.025 A), indicating that this medium has potential for prebiotic activity (Figure 7A). When the growth rates were compared in extracts from solid-state fermentation, the medium with glucose also stood out, with the most significant increase in OD, followed by JSC fermented by *Tyromices* sp. INPA1696 and CSC fermented by *P. lecomtei* BRM 044603, with OD variations of 0.954 ± 0.001 A and 0.922 ± 0.026 A, respectively (Figure 7B). All other fermented solids in both biomasses resulted in similar total cell growth of *L. plantarum* after 48 h of incubation.

**Figure 8:**
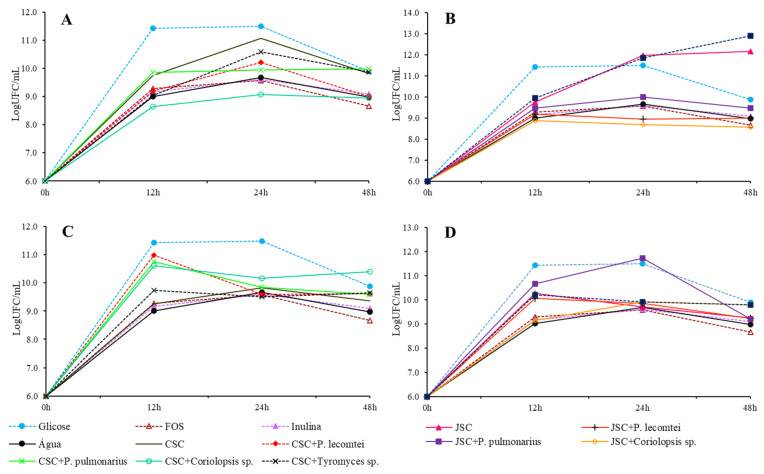
Growth of *L. plantarum* in LogUFC /mL with 0, 12, 24, and 48 h of incubation in MRS-C medium with 20 mg/mL of CSC submerged fermentation supernatants: (**A**), submerged fermentation supernatants of JSC (**B**), solid-state fermentation aqueous extracts of CSC (**C**), and solid-state fermentation extracts of JSC (**D**).

Among the treatments with fermented cakes, the highest growth was observed in the supernatants obtained from submerged fermentation of JSC by *P. pulmonarius* BRM 055674, while the lower results of total cell growth were observed in the supernatants obtained from *Coriolopsis* sp. INPA1646 fermentations (Table 4). The highest concentrations of viable cells were observed after 48 h of incubation in MRS media with 20 mg/mL of supernatants from submerged fermentation of JSC by *Tyromices* sp. INPA1696 and *P. pulmonarius* BRM 055674. It is important to note that after 48 h of incubation in these media, the concentration of viable cells seems to continue to rise, maintaining cell viability longer (Figure 8).

### 3.2. Antimicrobial Activity

Table 5 shows all the results of the antibiogram performed with supernatants (SmF) and aqueous extracts (SSF) lyophilized and diluted to concentrations of 100, 10, and 1 mg/mL. Strains were considered sensitive to aqueous extracts or supernatants when they presented inhibition halos greater than 10 mm and resistant when there was no halo formation or when it was smaller than 10 mm, as suggested by some authors [25,26,27]. Only the aqueous extracts from solid-state fermentation of CSC by *P. lecomtei* BRM 044603 and *Coriolopsis* sp. INPA1646 showed a halo of inhibition against *S. enterica* at a 100 mg/mL concentration. The optimization of the cultivation time to evaluate the kinetics of supernatants on *Salmonella* should be performed in a further study.

In addition to the aqueous extracts and supernatants from the CSC and JSC fermentations, the probiotic bacteria growth supernatants were also tested in an antibiogram assay for inhibition of enteropathogenic strains of *E. coli* ATCC25922, *S. aureus* ATCC27154, and *S. enterica* ATCC14028. During their growth, in addition to lactic acid and acetic acid, probiotic bacteria produce short-chain fatty acids and other extracellular metabolites that can inhibit the growth of some enterobacteria [28].

The *S. aureus* strain is more sensitive than the other two bacteria tested, as it showed inhibition halos for several *L. acidophilus* supernatants in the media with aqueous extracts of CSC fermented by *Tyromices* sp. INPA1696 and non-fermented JSC (Table 6). These results suggest that *L. acidophilus* releases some metabolic and antimicrobial activity in these strains of enteropathogens during its growth. 

The growth supernatants of *B. lactis* showed less inhibition of the enteropathogens than those of *L. acidophilus*. Table 6 shows the inhibition of supernatants of *B. lactis*, where it can be seen that only the supernatant of the growth of the probiotic with the supernatants (SmF) of JSC fermented by *P. lecomtei* BRM 044603 and *Coriolopsis* sp. INPA1646, CSC extracts without fungus, CSC fermented by *Tyromices* sp. INPA1696, JSC without fungus, and JSC fermented by *Coriolopsis* sp. INPA1646 can inhibit *S. aureus*, and only CSC extract fermented by *Tyromices* sp. INPA1696 and JSC without fungus were able to inhibit *S. enterica*.

The growth supernatants of *L. rhamnosus* have even less antibacterial activity on the strains tested, inhibiting only *S. aureus* and only when grown in the MRS with the CSC submerged fermentation supernatant (SmF) by *Coriolopsis* sp. INPA1646, and in the JSC supernatant without fungus. In *L. plantarum* supernatants, inhibition halos were not observed in any of the tested bacteria; because of that, the results of these supernatants do not appear in Table 6. Additionally, if any of the tested supernatants presented inhibition halos for *E. coli*, then just the results of *S. aureus* and *S. enterica* were shown in Table 6.

## 4. Discussion

*Coriolopsis* sp., *Tyromyces* sp., *P. lecomtei*, and *P. pulmonarius* were selected for this work because they showed the degradation activity of toxic compounds of plants such as gossypol and phorbol esters when grown by SSF in JSC (*Jatropha* seed cake) and CSC (cottonseed cake). 

In general, supernatants from submerged fermentations are better for the growth of *L. acidophilus* than the aqueous extracts tested, and the effect of fermentation with macrofungi *P. lecomtei* BRM 044603 and *Tyromyces* sp. INPA1696 increases the efficiency of extracts in stimulating the growth of *L. acidophilus*. In *B. lactis* cultures, our work corroborates Candela et al. (2010), when the commercial prebiotics FOS and inulin showed unsatisfactory growth [29]. However, Rodrigues et al. (2016) reported the growth of *Bifidobacterium* sp. higher in FOS than the negative control, with more significant growth after 24 h of incubation [23]. 

In fermented JSC, macrofungi’s growth reduces the efficiency of its supernatants for the growth of this bacteria by up to 70% under the evaluated cultivation conditions. Longer fermentation times can generate more fungal mass structures, such as chitin, beta-glucans, and mycosterols, among other chemical compounds, that can positively influence probiotic growth, such as *Ganoderma lucidum* polysaccharides, which increase the abundance of *Bifidobacteria*, *Lactobacillus*, *Roseburia*, *Lachnospiraceae*, and *Pleurotus eryngii*, increasing the proliferation of bacteria from the families *Porphyromonadaceae*, *Rikenellaceae*, *Bacteroidaceae*, and *Lactobacillaceae* [30]. Nonetheless, the 14th day fermentation supernatants showed less stimulus to *L. plantarum*, probably because the fungi used the readily assimilated carbohydrates in the biomass for their growth.

Other studies also pointed out the prebiotic potential of *Pleurotus* extracts. Synytsya et al. (2008) evaluated aqueous and alkaline extracts from *P. ostreatus* and *P. eryngii* for prebiotic activity by stimulating four *Lactobacillus* ssp., three *Bifidobacterium* ssp., and two *Enterococcus faecium* strains [31]. They observed that the aqueous extracts of both fungi stimulated *Lactobacillus* ssp., mainly *P. eryngii*. *Bifidobacterium* ssp. behaved differently, with one strain growing more with *P. ostreatus* extracts, another growing only with *P. eryngii* extracts, and a third one not stimulated by any extract. *Enterococcus faecium* strains grew less than all other strains in any of the extracts and showed better growth in alkaline extracts [31].

Ethanolic extracts of *P. ostreatus* (MTCC142) cultivated in solid and submerged states in banana crop residues showed antimicrobial activity against Gram-positive and Gram-negative bacteria and antifungal activity in vitro. Extracts from solid-state fermentation showed higher antibacterial activity against *Bacillus megaterium*, *Enterobacter*, and *P. aeruginosa* than those from submerged fermentation, more significant activity against *Micrococcus luteus* than standard antibiotics, and activity against *Enterobacter* and *P. aeruginosa* similar to that of chloramphenicol [32].

Some studies indicate that the antimicrobial activity of mushrooms occurs mainly against Gram-positive bacteria [18,33,34], which do not present the external lipid bilayer as in the Gram-negative ones, which may indicate that compounds with antimicrobial activity are water-soluble ions and are unable to cross a lipid bilayer. 

The metabolites produced by mushrooms and other fungi provide their extracts’ prebiotic and antimicrobial activities, as was observed in Clericuzio et al. (2021) [35]. In this work, metabolites were obtained by extraction using 90% acetone and 10% 2-propanol (*v*/*v*) from the fruiting bodies of 16 species of Basidiomycetes. Extracts were tested against pathogenic microorganisms by the disk diffusion technique. The results indicated that 12 of these species showed inhibition activity of *P. aeruginosa* ATCC 27853, mainly *Cortinarius nanceiensis*. However, none showed growth inhibition activity for *S. aureus* NCTC 6571, *K. pneumoniae* ATCC 13883, *C. albicans* ATCC 14053, or *C. glabrata* ATCC 15126. In our work, inhibition halos were observed in *S. aureus* with extracts of CSC fermented by *P. lecomtei* BRM 044603 and by *Coriolopsis* sp. INPA1646, indicating the great antimicrobial potential of these two extracts for pathogenic bacteria.

Some fungal exopolysaccharides obtained from aqueous extracts of Cerrena, Ganoderma, Lenzies, and Polyporus exhibited antimicrobial properties against Gram-positive and Gram-negative bacteria [2,36]. Demir and Yamac (2008) evaluated the antimicrobial activity of basidiocarp, submerged-growing mycelium, and exopolysaccharides from several basidiomycetes strains. Their results indicated that 91% of the 94 extracts showed activity against at least one tested microorganism, mainly *S. aureus*, *M. luteus*, and *E. faecium* [37].

In another study, the cultivation of *Coriolus versicolor* (*Trametes versicolor* (L.: Fr.)) for only 8 days in a bioreactor allowed the methanolic extraction of bioactive compounds with antimicrobial potential and high inhibitory activity for all Gram-positive bacteria tested (Duvnjak et al., 2016), mainly *Bacillus spizizeni*, *Staphylococcus epidermidis*, *Yersinia enterocolitica*, and six other Gram-negative bacterial strains from a total of ten tested. Extracellular polysaccharides were precipitated in the fermentation broth with ethanol and showed antibacterial activity against *Enterococcus faecalis* and *S. aureus* [38]. In this work, only aqueous extracts of CSC fermented by *Coriolopsis* sp. INPA1646 and *P. lecomtei* BRM 044603 inhibited *S. enterica* at the maximum concentration tested.

After extractions with ethyl acetate, acetone, and water, Chepkirui et al. (2018) isolated six compounds from the medicinal mushroom *Sanghuangporus* sp. three showed antimicrobial activity against *Micrococcus luteus*, and two of them showed non-selective antibacterial and antifungal activities [39]. Mahamat et al. (2018) performed the disc diffusion test and observed significant activity of *Termitomyces clypeatus* aqueous extract against *E. coli* ATCC 25922 (2.6 to 6.6 mm), *Enterobacter aerogenes* ATCC 13048 (1.8 to 2.8 mm), *S. aureus* MRSA (55 to 10.5 mm), *Salmonella typhi* Ty2 ATCC 700931 (0 to 5.5 mm), *C. albicans* ATCC10231 (3.9 to 8.5 mm), and *C. glabrata* ATCC 66032 (2.9 to 10.0 mm) [40], better results than those observed in this work for *E. coli* and *S. aureus*.

Although some supernatants have promoted significant inhibition halos in the antibiogram assays, mainly against *S. aureus*, it was impossible to perform MIC (minimum inhibitory concentration) and MBC (minimum bactericidal concentration) assays. The exact concentration of these lactic fermentation supernatants is unknown, as they are the medium MRS, supplemented or not, with the extracellular metabolites of the tested probiotic bacteria. Optimizing macrofungal cultivation to obtain supernatants and crude extracts as carbon sources for probiotic bacteria is a step to be studied further to seek better results against enteropathogenic bacteria.

## 5. Conclusions

Submerged or solid-state fermentation methods and obtaining liquid extracts can alter probiotic bacteria’s growth and inhibit enteropathogenic bacteria. Unfermented JSC supernatant showed better results for the growth of four bacterial strains than other treatments, indicating that *Jatropha* cake, in nature, contains metabolites or carbohydrate benefits for probiotic bacteria. Supernatants and aqueous extracts obtained from CSC fermentation can be used in culture media for probiotic bacteria, mainly *L. acidophilus*, and antimicrobials obtained from solid-state fermentation with *P. lecomtei* BRM 044603 and *Coriolopsis* sp. INPA1646. The chemical characteristics of these extracts can clarify the mechanism of probiotic and antimicrobial biological activities, so it is necessary to explore secretory analysis in future studies and evaluate the best conditions and combinations of these extracts. In the future, the best extract needs to have clarified mechanisms of antimicrobial activity through metabolomic analyses, in vivo trials, and evaluation of its effect on enteropathogens from the rumen.

## Figures and Tables

**Table 1:** Total cell growth of *L. acidophilus* (OD 600) in 48 h of incubation (mean ± SD) in MRS medium with 20 mg/mL of the different supernatants from submerged cultures and aqueous extracts from solid-state fermentation using macrofungi grown in JSC and CSC.

Type of Fermentation	Fermented Biomass	Variation of OD (Tf-Ti *)
Control (C−)	Water	0.564 ± 0.028 ^j^
Controls (C+)	Glucose	2.081 ± 0.017 ^b^
	FOS	0.476 ± 0.031 ^k^
	Inulin	0.432 ± 0.026 ^k^
Supernatants of submerged fermentations	CSC	0.630 ± 0.026 ^i^
	CSC + *P. lecomtei*	1.383 ± 0.028 ^c^
	CSC + *P. pulmonarius*	1.139 ± 0.018 ^d^
	CSC + *Coriolopsis* sp.	0.922 ± 0.009 ^e^
	CSC + *Tyromyces* sp.	1.217 ± 0.005 ^d^
Aqueous extracts of solid-state fermentations	CSC	0.720 ± 0.056 ^h^
	CSC + *P. lecomtei*	0.991 ± 0.026 ^e^
	CSC + *P. pulmonarius*	0.865 ± 0.026 ^f^
	CSC + *Coriolopsis* sp.	0.851 ± 0.007 ^f^
	CSC + *Tyromyces* sp.	1.203 ± 0.058 ^d^
Supernatants of submerged fermentations	JSC	2.139 ± 0.022 ^a^
	JSC + *P. lecomtei*	0.849 ± 0.053 ^f^
	JSC + *P. pulmonarius*	1.022 ± 0.015 ^e^
	JSC + *Coriolopsis* sp.	0.774 ± 0.028 ^h^
	JSC + *Tyromyces* sp.	0.960 ± 0.058 ^e^
Aqueous extracts of solid-state fermentations	JSC	0.107 ± 0.046 ^l^
	JSC + *P. lecomtei*	0.816 ± 0.001 ^g^
	JSC + *P. pulmonarius*	0.748 ± 0.062 ^g^
	JSC + *Coriolopsis* sp.	0.764 ± 0.008 ^g^
	JSC + *Tyromyces* sp.	0.861 ± 0.019 ^f^

* Tf-Ti = difference between final absorbance (after 48 h of incubation) and initial absorbance. Different letters indicate statistically significant differences using Tukey’s test (*p* < 0.05).

**Table 2:** Growth of total *B. lactis* cells (OD 600) in 48 h of incubation (mean ± SD) in MRS medium with 20 mg/mL of different supernatants from submerged cultures and aqueous extracts from solid-state fermentation using macrofungi grown in JSC and CSC.

Type of Fermentation	Fermented Biomass	Variation of OD (Tf-Ti *)
Control (C−)	Water	0.303 ± 0.041 ^i^
Controls (C+)	Glucose	2.122 ± 0.086 ^b^
	FOS	0.735 ± 0.077 ^e^
	Inulin	0.745 ± 0.120 ^e^
Supernatants of submerged fermentation	CSC	0.907 ± 0.012 ^d^
	CSC + *P. lecomtei*	0.654 ± 0.003 ^e^
	CSC + *P. pulmonarius*	0.949 ± 0.059 ^d^
	CSC + *Coriolopsis* sp.	0.379 ± 0.007 ^h^
	CSC + *Tyromyces* sp.	0.627 ± 0.021 ^f^
Aqueous extracts of solid-state fermentation	CSC	0.833 ± 0.020 ^e^
	CSC + *P. lecomtei*	0.906 ± 0.001 ^d^
	CSC + *P. pulmonarius*	0.888 ± 0.015 ^d^
	CSC + *Coriolopsis* sp.	0.728 ± 0.005 ^e^
	CSC + *Tyromyces* sp.	1.318 ± 0.087 ^c^
Supernatants of submerged fermentation	JSC	2.501 ± 0.140 ^a^
	JSC + *P. lecomtei*	0.845 ± 0.052 ^d^
	JSC + *P. pulmonarius*	0.817 ± 0.020 ^d^
	JSC + *Coriolopsis* sp.	0.272 ± 0.027 ^i^
	JSC + *Tyromyces* sp.	0.478 ± 0.067 ^g^
Aqueous extracts of solid-state fermentation	JSC	0.126 ± 0.020 ^j^
	JSC + *P. lecomtei*	1.366 ± 0.034 ^c^
	JSC + *P. pulmonarius*	1.295 ± 0.017 ^c^
	JSC + *Coriolopsis* sp.	0.906 ± 0.140 ^d^
	JSC + *Tyromyces* sp.	1.342 ± 0.022 ^c^

* Tf-Ti = difference between final absorbance (after 48 h of incubation) and initial absorbance. Different letters indicate statistically significant differences using Tukey’s test (*p* < 0.05).

**Table 3:** Total cell growth of *L. rhamnosus* (OD 600) in 48 h of incubation (mean ± SD) in MRS medium with 20 mg/mL of the different supernatants from submerged cultures and aqueous extracts from solid state fermentations using macrofungi grown in JSC and CSC.

Type of Fermentation	Fermented Biomass	Variation of OD (Tf-Ti *)
Control (C−)	Water	0.438 ± 0.113 ^h^
Controls (C+)	Glucose	2.279 ± 0.025 ^b^
	FOS	0.137 ± 0.126 ^i^
	Inulin	0.677 ± 0.046 ^f^
Supernatants of submerged fermentation	CSC	0.881 ± 0.059 ^ef^
	CSC + *P. lecomtei*	0.481 ± 0.003 ^fg^
	CSC + *P. pulmonarius*	0.825 ± 0.002 ^e^
	CSC + *Coriolopsis* sp.	0.404 ± 0.081 ^fg^
	CSC + *Tyromyces* sp.	0.636 ± 0.038 ^f^
Aqueous extracts of solid-state fermentation	CSC	0.913 ± 0.089 ^cd^
	CSC + *P. lecomtei*	0.802 ± 0.003 ^f^
	CSC + *P. pulmonarius*	0.965 ± 0.104 ^de^
	CSC + *Coriolopsis* sp.	0.825 ± 0.137 ^ef^
	CSC + *Tyromyces* sp.	1.199 ± 0.091 ^c^
Supernatants of submerged fermentation	JSC	2.570 ± 0.057 ^a^
	JSC + *P. lecomtei*	0.641 ± 0.025 ^f^
	JSC + *P. pulmonarius*	0.766 ± 0.005 ^e^
	JSC + *Coriolopsis* sp.	0.302 ± 0.032 ^h^
	JSC + *Tyromyces* sp.	0.512 ± 0.042 ^g^
Aqueous extracts of solid-state fermentation	JSC	0.397 ± 0.091 ^h^
	JSC + *P. lecomtei*	0.725 ± 0.029 ^f^
	JSC + *P. pulmonarius*	1.042 ± 0.049 ^d^
	JSC + *Coriolopsis* sp.	0.922 ± 0.051 ^e^
	JSC + *Tyromyces* sp.	0.920 ± 0.026 ^e^

* Tf-Ti = difference between final absorbance (after 48 h of incubation) and initial absorbance. Different letters indicate statistically significant differences using Tukey’s test (*p* < 0.05).

**Table 4:** Total cell growth of *L. plantarum* (OD 600) in 48 h of incubation (mean ± SD) in MRS medium with 20 mg/mL of different submerged culture supernatants and aqueous extracts of macrofungal solid-state fermentations of JSC and CSC.

Type of Fermentation	Fermented Biomass	Variation of OD (Tf-Ti *)
Control (C−)	Water	0.462 ± 0.105 ^f^
Controls (C+)	Glucose	2.014 ± 0.018 ^a^
	FOS	0.370 ± 0.041 ^f^
	Inulin	0.351 ± 0.017 ^f^
Supernatants of submerged fermentation	CSC	0.728 ± 0.023 ^d^
	CSC + *P. lecomtei*	0.862 ± 0.018 ^c^
	CSC + *P. pulmonarius*	0.965 ± 0.048 ^b^
	CSC + *Coriolopsis* sp.	0.578 ± 0.107 ^e^
	CSC + *Tyromyces* sp.	0.931 ± 0.011 ^b^
Aqueous extracts of solid-state fermentation	CSC	0.601 ± 0.033 ^e^
	CSC + *P. lecomtei*	0.922 ± 0.026 ^b^
	CSC + *P. pulmonarius*	0.805 ± 0.016 ^d^
	CSC + *Coriolopsis* sp.	0.779 ± 0.022 ^d^
	CSC + *Tyromyces* sp.	0.776 ± 0.058 ^d^
Supernatants of submerged fermentation	JSC	2.026 ± 0.025 ^a^
	JSC + *P. lecomtei*	0.723 ± 0.039 ^d^
	JSC + *P. pulmonarius*	0.881 ± 0.054 ^bc^
	JSC + *Coriolopsis* sp.	0.593 ± 0.095 ^e^
	JSC + *Tyromyces* sp.	0.706 ± 0.033 ^d^
Aqueous extracts of solid-state fermentation	JSC	0.329 ± 0.030 ^f^
	JSC + *P. lecomtei*	0.724 ± 0.017 ^d^
	JSC + *P. pulmonarius*	0.727 ± 0.019 ^d^
	JSC + *Coriolopsis* sp.	0.729 ± 0.044 ^d^
	JSC + *Tyromyces* sp.	0.954 ± 0.001 ^b^

* Tf-Ti = difference between final absorbance (after 48 h of incubation) and initial absorbance. Different letters indicate statistically significant differences using Tukey’s test (*p* < 0.05).

**Table 5:** Results of antibiogram tests to evaluate inhibition halos of *E. coli*, *S. aureus*, and *S. enterica* strains when treated with submerged fermentation supernatants (SmF) and aqueous extracts from solid state fermentations (SSF) of macrofungi in CSC and JSC.

Treatment	Inhibition Halo Diameter								
	*E. coli*			*S. aureus*			*S. enterica*		
	100 mg/mL	10 mg/mL	1 mg/mL	100 mg/mL	10 mg/mL	1 mg/mL	100 mg/mL	10 mg/mL	1 mg/mL
Water	-	-	-	-	-	-	-	-	-
CSC (SmF)	-	-	-	-	-	-	-	-	-
CSC + BRM 044603 (SmF)	-	-	-	-	-	-	-	-	-
CSC + BRM 055674 (SmF)	-	-	-	-	-	-	-	-	-
CSC + INPA1646 (SmF)	-	-	-	-	-	-	-	-	-
CSC + INPA1696 (SmF)	-	-	-	-	-	-	-	-	-
JSC (SmF)	-	-	-	-	-	-	-	-	-
JSC + BRM 044603 (SmF)	-	-	-	-	-	-	-	-	-
JSC + BRM 055674 (SmF)	-	-	-	-	-	-	-	-	-
JSC + INPA1646 (SmF)	-	-	-	-	-	-	-	-	-
JSC + INPA1696 (SmF)	-	-	-	-	-	-	-	-	-
CSC (SSF)	-	-	-	-	-	-	-	-	-
CSC + BRM 044603 (SSF)	-	-	-	-	-	-	12 mm	-	-
CSC + BRM 055674 (SSF)	-	-	-	-	-	-	-	-	-
CSC + INPA1646 (SSF)	-	-	-	-	-	-	7.3 mm	-	-
CSC + INPA1696 (SSF)	-	-	-	-	-	-		-	-
JSC (SSF)	-	-	-	-	-	-	-	-	-
JSC + BRM 044603 (SSF)	-	-	-	-	-	-	-	-	-
JSC + BRM 055674 (SSF)	-	-	-	-	-	-	-	-	-
JSC + INPA1646 (SSF)	-	-	-	-	-	-	-	-	-
JSC + INPA1696 (SSF)	-	-	-	-	-	-	-	-	-
Antibiotic (C+)	21 mm			37.5 mm			36 mm		

**Table 6:** Results of antibiogram assays to assess halos of inhibition of *E. coli*, *S. aureus*, and *S. enterica* strains by probiotic bacteria growth supernatants in the samples tested in the prebiotic activity assays.

	*L. acidophilus* Supernatants			*B. lactis* Supernatants			*L. rhamnosus* Supernatants		
	*E. coli*	*S. aureus*	*S. enterica*	*E. coli*	*S. aureus*	*S. enterica*	*E. coli*	*S. aureus*	*S. enterica*
Glucose	-	9.5 mm	-	-	-	-	-	-	-
FOS	-	9 mm	-	-	-	-	-	-	-
Inulin	-	7 mm	-	-	-	-	-	-	-
Water	-	-	-	-	-	-	-	-	-
CSC (SmF)	-	-	-	-	-	-	-	-	-
CSC + BRM 044603 (SmF)	-	-	-	-	-	-	-	-	-
CSC + BRM 055674 (SmF)	-	-	-	-	-	-	-	-	-
CSC + INPA1646 (SmF)	-	8 mm	-	-	-	-	-	13 mm	-
CSC + INPA1696 (SmF)	-	7 mm	-	-	-	-	-	-	-
JSC (SmF)	-	10 mm	-	-	-	-	-	10 mm	-
JSC + BRM 044603 (SmF)	-	8 mm	-	-	7 mm	-	-	-	-
JSC + BRM 055674 (SmF)	-	10 mm	-	-	-	-	-	-	-
JSC + INPA1646 (SmF)	-	10 mm	-	-	10.5 mm	-	-	-	-
JSC + INPA1696 (SmF)	-	-	-	-	-	-	-	-	-
CSC (SSF)	-	11 mm	-	-	9.5 mm	-	-	-	-
CSC + BRM 044603 (SSF)	-	-	-	-	-	-	-	-	-
CSC + BRM 055674 (SSF)	-	-	-	-	-	-	-	-	-
CSC + INPA1646 (SSF)	-	-	-	-	-	-	-	-	-
CSC + INPA1696 (SSF)	-	-	14 mm	-	11.5 mm	14 mm	-	-	-
JSC (SSF)	-	9 mm	13 mm	-	8 mm	13 mm	-	-	-
JSC + BRM 044603 (SSF)	-	-	-	-	-	-	-	-	-
JSC + BRM 055674 (SSF)	-	-	-	-	-	-	-	-	-
JSC + INPA1646 (SSF)	-	10 mm	-	-	12 mm	-	-	-	-
JSS + INPA1696 (SSF)	-	-	-	-	-	-	-	-	-
Antibiotic (C+)	21 mm	37.5 mm	36 mm	21 mm	37.5 mm	36 mm	21 mm	37.5 mm	36 mm

## Data Availability

The data presented in this study are available in article.
